# Estrogen/Estrogen Receptor Alpha Signaling in Mouse Posterofrontal Cranial Suture Fusion

**DOI:** 10.1371/journal.pone.0007120

**Published:** 2009-09-22

**Authors:** Aaron W. James, Alexander A. Theologis, Samantha A. Brugmann, Yue Xu, Antoine L. Carre, Philipp Leucht, Katherine Hamilton, Kenneth S. Korach, Michael T. Longaker

**Affiliations:** 1 Hagey Pediatric Regenerative Research Laboratory, Department of Surgery, Division of Plastic and Reconstructive Surgery, Stanford University School of Medicine, Stanford, California, United States of America; 2 School of Medicine, University of California San Francisco, San Francisco, California, United States of America; 3 Department of Orthopaedic Surgery, Stanford University School of Medicine, Stanford, California, United States of America; 4 Receptor Biology Section, Laboratory of Reproductive and Developmental Toxicology, National Institute of Environmental Health Sciences, National Institutes of Health, Research, Triangle Park, North Carolina, United States of America; Ecole Normale Supérieure de Lyon, France

## Abstract

**Background:**

While premature suture fusion, or craniosynostosis, is a relatively common condition, the cause is often unknown. Estrogens are associated with growth plate fusion of endochondral bones. In the following study, we explore the previously unknown significance of estrogen/estrogen receptor signaling in cranial suture biology.

**Methodology/Principal Findings:**

Firstly, estrogen receptor (ER) expression was examined in physiologically fusing (posterofrontal) and patent (sagittal) mouse cranial sutures by quantitative RT-PCR. Next, the cranial suture phenotype of ER alpha and ER beta knockout (αERKO, βERKO) mice was studied. Subsequently, mouse suture-derived mesenchymal cells (SMCs) were isolated; the effects of 17-β estradiol or the estrogen antagonist Fulvestrant on gene expression, osteogenic and chondrogenic differentiation were examined *in vitro*. Finally, *in vivo* experiments were performed in which Fulvestrant was administered subcutaneously to the mouse calvaria. Results showed that increased *ERα* but not *ERβ* transcript abundance temporally coincided with posterofrontal suture fusion. The αERKO but not βERKO mouse exhibited delayed posterofrontal suture fusion. *In vitro*, addition of 17-β estradiol enhanced both osteogenic and chondrogenic differentiation in suture-derived mesenchymal cells, effects reversible by Fulvestrant. Finally, *in vivo* application of Fulvestrant significantly diminished calvarial osteogenesis, inhibiting suture fusion.

**Conclusions/Significance:**

Estrogen signaling through ERα but not ERβ is associated with and necessary for normal mouse posterofrontal suture fusion. *In vitro* studies suggest that estrogens may play a role in osteoblast and/or chondrocyte differentiation within the cranial suture complex.

## Introduction

Craniosynostosis, or the premature osseous obliteration of cranial sutures, is a relatively common craniofacial defect, with an incidence of 1 in 2500 live births [1]. Its clinical sequelae can be significant, and include both morphologic and functional abnormalities [2]. Our laboratory has investigated the mechanisms of cranial suture fusion using a mouse model, with the hopes that a more thorough understanding of physiologic suture fusion may lead toward novel therapeutics for pathologic synostosis [3].

In the mouse, the posterofrontal (PF) suture fuses in the first weeks of life, whereas all other sutures remain patent, including the contiguous sagittal (SAG) suture [3], [4]. The PF suture normally fuses through a cartilage intermediate, or via endochondral ossification [4]. In these respects, the mouse PF suture is analogous to the human metopic suture, which fuses during infancy [5], [6]. The molecular comparison of PF and SAG cranial sutures, as exemplars of normal suture fusion and suture patency, respectively, has led to the identification of differences in locally acting cytokines between fusing and patent sutures, including FGF-2, IGFs, TGF-βs, and BMP agonists and antagonists [7]–[11]. While much study has focused on the role of autocrine and paracrine signaling in cranial suture biology, the role of endocrine signaling in suture formation and fusion has been relatively unexplored [12], [13].

Estrogens have been shown to be important in the development and maintenance of the appendicular skeleton [14]. Estrogens have two known nuclear receptors, estrogen receptor (ER) α and β. Both receptors are present in the epiphyseal growth plate, specifically in hypertrophic chondrocytes, as well as adjacent bony tissues [15]–[18]. The mechanisms by which estrogens act locally on the growth plate are poorly understood. It has been proposed that estrogen initiates the pubertal growth spurt by stimulating chondrogenesis and inhibiting chondrocyte apoptosis [19]. Additionally, estrogens are postulated to contribute to growth plate fusion via endochondral ossification, possibly by estrogen-induced vasculogenesis and/or osteoblast invasion [20], [21].

Despite the imprecise role of estrogen in growth plate fusion, perturbations of estrogen signaling indicate its importance. Both the ERα and ERβ knockout mice (αERKO and βERKO) possess a skeletal phenotype. The αERKO mouse has been observed to have a more severe phenotype, including reduced bone mineral density and bone mineral content, as well as decreased longitudinal and radial bone growth [14], [22]–[26]. Absence of the tibial growth plate cartilages has also been previously observed in female αERKO mice [26]. In contrast, the βERKO mouse has been observed to have a relatively minor phenotype, reported either to have normal or increased appendicular and axial-skeletal growth [24], [27], [28]. Human perturbations in estrogen signaling have been observed to differ from those in mice: patients with mutations in ERα or aromatase P-450 present with delayed or incomplete ossification of the growth plates [29]–[31]. While estrogen's involvement in longitudinal bone growth has been studied in depth, its role in cranial suture biology has not been defined. We hypothesized that estrogen may influence the PF suture in a similar manner to its role in ossification of the epiphyseal plate.

In this study, we first examined temporal and spatial patterns of ER expression in the posterofrontal (PF, fusing) and sagittal (SAG, patent) sutures. Next, we inquired as to the necessity of estrogen signaling in cranial suture fusion by examining the suture phenotype of the αERKO and βERKO mutant mice. We employed *in vitro* culture of calvarial-derived cells to explore in detail the cellular impacts of estrogen signaling, utilizing both 17-β estradiol (E2) and the pan-ER antagonist Fulvestrant (ICI 182,780). Finally, *in vivo* biochemical perturbations were performed by applying Fulvestrant to developing wild-type cranial sutures.

## Results

### Gross and histological morphology of Posterofrontal and Sagittal cranial sutures

Our laboratory has previously identified a timeline of normal PF suture fusion: the initiation of fusion occurs at or around postnatal day 7 (p7), the most prominent cartilaginous intermediate is observed at p10, and osseous closure is for the most part complete by p19 [4]. We sought to confirm this by whole mount bone and cartilage preparations of mouse calvariae, (Fig. S1). In the murine skull, the PF suture lies anterior to the contiguous SAG suture, which lies posterior (Fig. S1A). At p4, the calvarial bones are widely separated by suture mesenchyme (Fig. S1A). Intermittent bony bridging of the PF suture is apparent at or around p10, primarily in the anterior aspect of the suture (Fig. S1C). By p19, the majority of the PF suture has undergone osseous fusion, with intermittent gaps of patency (Fig. S1F). In contrast, the SAG suture remains patent in its entirety, throughout adult life (Fig. S1A–F).

Histological analysis of PF and SAG sutures reveals more developmental detail, (Fig. S2). Pentachrome staining was performed at identical time points (p4–p19), in which bone appears yellow, while cartilage appears blue/green. As the histologic appearance of PF and SAG sutures exhibits variation along the anterior-posterior axis, representative sections from the anterior aspect of each suture were chosen. At p4, the osteogenic fronts of both PF and SAG sutures are widely separated by an undifferentiated cellular mesenchyme, (Fig. S2A,B,G,H). From p7 to p10, a blue/green cartilaginous intermediate appears on the endocranial aspect of the PF suture (Fig. S2C-F). At p16 onwards, the endocranial mesenchyme of the PF suture is replaced by bony tissue seen as yellow (i.e. fused) (Fig. S2O-R). In contrast, the osteogenic fronts of the SAG suture remain separated by a cellular mesenchyme at all time points (i.e. patent), and either appear in an end-to-end or slightly overlapping fashion (Fig. S2G–L,S–X).

### Estrogen receptor gene expression in PF and SAG cranial sutures

**Figure 1:**
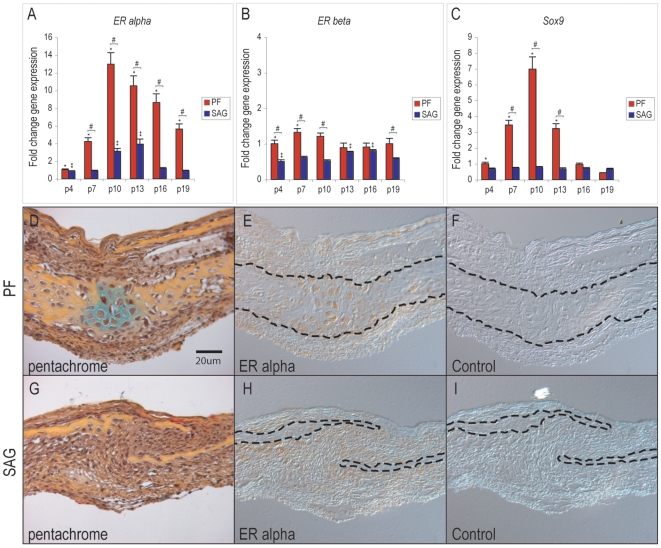
Estrogen receptor α and β expression in PF and SAG sutures. (A) *ERα* gene expression in PF and SAG sutures by qRT-PCR, normalized to *GAPDH*. Expression increased over 12-fold from postnatal ages 4 to 10 in the fusing PF suture, see red bars. Highest expression was noted at p10, temporally corresponding with PF suture fusion. (B) *ERβ* gene expression in PF and SAG sutures, normalized to *GAPDH*. Expression remained relatively constant over the time course examined in both PF and SAG sutures. (C) *Sox9* gene expression in PF and SAG sutures, normalized to *GAPDH*. (D) Pentachrome staining of the p7 PF suture in which cartilage appears blue/green, while bone appears yellow. (E) ERα immunohistochemistry of p7 PF suture. Nuclear ERα protein strongly localizes to hypertrophic chondrocytes within the PF suture; dashed lines indicate the fusing inner bone table of the suture. (F) Negative control for ERα immunohistochemistry. (G) Pentachrome staining of the p7 SAG suture. (H) ERα immunohistochemistry of p7 SAG suture. Dashed lines indicate the adjacent osteogenic fronts of the patent suture. (I) Negative control for ERα immunohistochemistry. Gene expression values are normalized to expression within the p4 SAG suture complex, N = 10, *, **, and #*P≤*0.01 in which * signifies differences between the PF suture at various timepoints, ** between SAG suture at various timepoints, and # between PF and SAG sutures at the same timepoint. Histological sections are from the anterior aspect of PF and SAG sutures, presented at 40× magnification.

Having confirmed a timeline for PF suture fusion, estrogen receptor expression was examined by qRT-PCR. Expression of both nuclear receptors, *ERα* and *ERβ*, was assayed in PF (fusing) as well as SAG (patent) cranial sutures, (Fig. 1A,B). Results showed that *ERα* transcript abundance in the PF suture increased over the time course of suture fusion, (red bars, Fig. 1A). Before the process of suture fusion (p4), *ERα* expression was approximately equal between PF and SAG sutures (far left, Fig. 1A, N = 10). From p4 to p10, a greater than 12-fold increase in *ERα* was observed, (corresponding to a time period of growth and chondrogenesis within the PF suture, (red bars Fig. 1A, *P<0.01). Thereafter, *ERα* expression gradually decreased, but remained significantly elevated, (a time period corresponding to gradual suture ossification, red bars Fig. 1A, *P<0.01). *ERα* expression was present to a lesser degree in the non-fusing SAG suture, (blue bars Fig. 1A, #*P*<0.01). While *ERα* expression peaked during fusion of the PF suture, *ERβ* expression remained relatively constant overtime in both PF and SAG suture complexes, (Fig. 1B). Expression of the chondrogenic transcription factor *Sex determining region Y-box 9* (*Sox9*) was also evaluated, (Fig. 1C). The peak in transcript abundance for both *Sox9* and *ERα* temporally coincided at p10, a time point at which histological evidence of chondrocytes is most apparent in the PF suture, (Fig. 1C, see also Supplemental Fig. 2,E,F, N, *P<0.01). Data of *Sox9* expression are in agreement with previous observations by our laboratory [4]. In summary, these data temporally associated ERα gene expression with mouse PF suture fusion.

### Estrogen receptor protein expression in PF and SAG cranial sutures

Having observed a dynamic change in *ERα* gene expression, we next examined the spatial distribution of ERα protein expression in the fusing PF and patent SAG sutures, by immunohistochemistry (Fig. 1D–I). In the PF suture, strong nuclear staining for ERα was observed most prominently in hypertrophic chondrocytes in the suture mesenchyme, (Fig. 1E). Less prominent staining was observed within the pericranium, as well as osteoblasts lining and osteocytes within the frontal bones. For comparison in the SAG suture, immunostaining for ERα protein was limited to those cells encircling the osteogenic fronts of the growing parietal bones, (Fig. 1H). Pentachrome staining of adjacent sections are provided for orientation (Fig. 1D,G); negative controls without exposure to primary antibody are presented as well (Fig. 1F,I). Notably, no significant differences in staining for ERs were observed between sutures taken from male or female mice, a finding similar to previously published observation in the murine tiba [15], [16]. In summary, ERα protein is expressed in both PF and SAG sutures. Its spatial distribution suggests that cells undergoing both osteogenesis and chondrogenesis within the developing cranial suture expressed ER protein. In addition, cells within the suture mesenchyme of the non-fusing SAG suture stained to a relatively lesser degree for ER protein than chondrocytes within the fusing PF suture.

### ERα but not ERβ null mice exhibit delayed PF suture fusion

**Figure 2:**
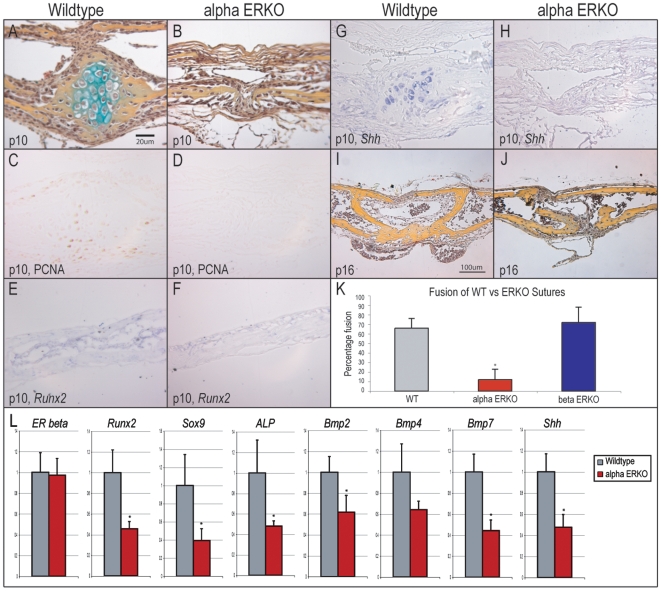
PF suture phenotype in αERKO mouse. (A) Wild-type (WT) p10 PF suture. Hypertrophic chondrocytes, seen here as blue/green, are observed to bridge the gap between osteogenic fronts of the suture. (B) αERKO p10 PF suture. A complete absence of cartilage is observed, despite proximity of the osteogenic fronts. (C) PCNA immunohistochemistry in the WT p10 PF suture. (D) PCNA staining in the αERKO p10 PF suture showed relatively less staining. (E) *In situ* hybridization for *Runx2* in the WT p10 frontal bone. Strong staining was noted in the cells lining and within the bone. (F) In comparison, less staining was observed in the αERKO frontal bone (G) *In situ* hybridization for *Shh* in the WT p10 PF suture. Strong staining was noted in the hypertrophic chondrocytes within the suture mesenchyme. (H) In comparison, little staining for *Shh* was noted in the αERKO p10 PF suture. (I) WT p16 PF suture. Note normal osseous fusion on the endocranial aspect. (J) αERKO p16 PF suture. Suture patency is apparent on both endo- and ectocranial aspects. (K) Mean percentage suture fusion in WT, αERKO and βERKO PF p16 sutures. Every 5^th^ coronal section of the suture was stained; two blinded, independent observers judged fusion or patency along the length of the suture. (L) Relative gene expression in WT as compared to αERKO whole skulls at 7d of life, as determined by qRT-PCR. Averages and standard deviations were calculated, significance calculated relative to wild-type percentage fusion, *P<0.01. For histological specimens, sections presented are taken from the anterior aspect of the PF suture, presented at 20×–40× magnification.

While ER expression was associated with PF suture fusion, the necessity of ER signaling in suture fusion had not yet been assessed. For this purpose, genetic knockout mice were analyzed lacking either functional ERα or ERβ. Analysis of the PF suture was performed at postnatal time points spanning the process of suture fusion: p10, p16 and p30. As the PF suture exhibits variation in development along its anterior-posterior axis, the entirety of each suture was sectioned and every 5^th^ section was stained for histological analysis (Fig. 2).

In the p10 PF wild-type (WT) suture a large cartilaginous intermediate was normally observed within the mid-sutural space (Fig. 2A). Cartilage was observed in the majority of sections, and found in all WT sutures (5/5 specimens). In the corresponding αERKO sutures, the PF suture was observed to be patent throughout and without a cartilage intermediate, (Fig. 2B) (3/3 specimens). Cellular proliferation was assayed in WT and αERKO by PCNA immunostaining, which showed a relative paucity of PCNA+ cells in the knockout PF suture (Fig. 2C,D). Further difference in gene expression were evaluated by *in situ* hybridization (Fig. 2E–H). In the WT frontal bone, those cells lining and within the calvarial bone stained strongly for *Runx2*, while relatively less staining was observed in the αERKO frontal bone (Fig. 2E,F). Moreover, in the WT PF suture, hypertrophic chondrocytes were noted to stain strongly for *Sonic Hedgehog* (*Shh*), by *in situ* hybridization (Fig. 2G). In contrast, little *Shh* expression was observed in the αERKO PF suture (Fig. 2H). By postnatal day 16, the majority of the WT PF suture exhibited osseous fusion on the endocranial surface, (Fig. 2I). In contrast, only sporadic fusion was noted in the αERKO PF suture, (Fig. 2J). Percentage fusion was calculated by examination of every 5^th^ slide (N = 5 WT, N = 3 αERKO sutures). Results showed that the majority of the WT PF suture exhibited fusion along its anterior-posterior axis by p16 (66%), whereas on average only 12% of the αERKO PF suture was fused along its length (Fig. 2K, *P<0.01). This difference in PF fusion was observed in 30d old mice as well: while osseous fusion was apparent throughout the length of the PF suture in the WT and with only small gaps of patency (N = 4), only the anterior portion of αERKO PF suture showed evidence of fusion and osteogenic fronts remained separated (data not shown). Analysis of the βERKO PF suture revealed no difference to WT sutures (Fig. 2K for quantization). Moreover, no differences in SAG suture morphology were observed between wild-type, αERKO, or βERKO mice (data not shown). Collectively, these data demonstrated that the αERKO but not the βERKO mouse exhibited delayed fusion of the PF suture, without evidence of a chondrocytic intermediate and with diminished Hedgehog expression.

To verify differences between wild-type and αERKO mice observed by histology, gene expression in whole skulls was examined in 7d old littermates. Briefly, numerous genes involved in osteochondrogenic differentiation were observed to be decreased in expression in the αERKO calvaria, including *Runx2, Sox9* and *alkaline phosphatase (ALP)* (Fig. 2L). Moreover, growth and differentiation factors such as *BMP-2, -4*, and -*7*, as well as *Sonic Hedgehog* were down-regulated as well (Fig. 2L, right).

While ERα mutations resulted in a mouse suture phenotype, the underlying mechanisms that translated a biochemical deficiency into a craniofacial dysmorphism remained unclear. To elucidate possible mechanisms, we next turned to *in vitro* culture of suture-derived mesenchymal cells (SMCs), of which the procedure has been previously described [7], [8], [32]. *In vitro*, estrogen signaling was either artificially increased with the addition of 17-β estradiol (E2), or decreased with addition of the synthetic, pan-ER antagonist Fulvestrant (ICI 182,780).

### 17-β Estradiol increases cellular proliferation of PF suture-derived mesenchymal cells, reversible by Fulvestrant

**Figure 3:**
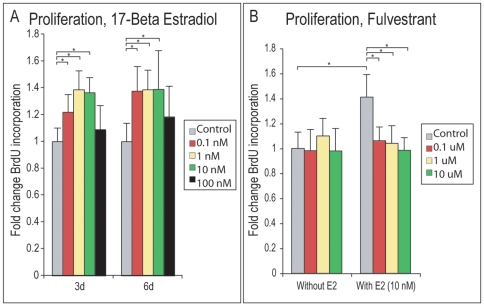
Cellular proliferation of PF SMCs with 17-β Estradiol and Fulvestrant. After 3 and 6d growth with or without 17-β estradiol (0.1–100 nM), BrdU incorporation assays were performed to evaluate cellular proliferation. (A) BrdU incorporation with or without 17-β estradiol. Treatment with 17-β estradiol (E2) resulted in increased proliferation (0.1–10 nM). (B) BrdU incorporation after 6d growth with Fulvestrant alone or in combination with 17-β estradiol. Fulvestrant alone showed no effect on BrdU uptake (left), while in combination with E2 was observed to reverse the mitogenic effect of E2 (right). Values are normalized and significance levels calculated relative to control groups in grey, N = 6, *P<0.01.

Posterofrontal suture-derived mesenchymal cells (SMCs) were isolated from p4 animals, a time period where the PF suture is widely patent, separated by an undifferentiated cellular mesenchyme (see also Supplemental Fig. 2A,B for histology). To assess the effects of estrogen signaling in SMCs, both E2 and the ER antagonist Fulvestrant were added to medium, alone or in combination; *in vitro* studies included proliferation, osteogenic and chondrogenic differentiation assays. Effects on cellular proliferation were first examined by BrdU incorporation assays (Fig. 3). At both 3 and 6d growth, E2 was observed to increase BrdU incorporation, (Fig. 3A, 0.1–10 nM, N = 6, *P<0.01). No effect was observed with blockade of estrogen signaling via Fulvestrant addition to medium alone (left Fig. 3B, 0.1–10 uM). By combining Fulvestrant with E2, the mitogenic effect of E2 was reversed, (right Fig. 3B, 10 nM estradiol, 0.1–10 uM Fulvestrant, *P<0.01).

### 17-β Estradiol increases osteogenic differentiation of PF suture-derived mesenchymal cells

**Figure 4:**
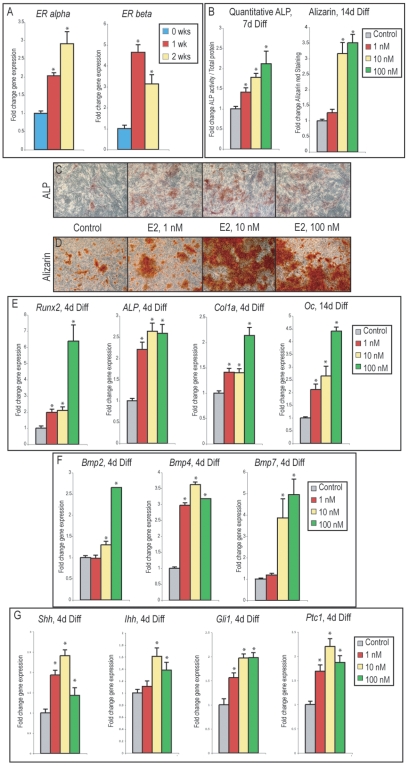
Osteogenic differentiation of PF SMCs with 17-β Estradiol. (A) *ERα* and *ERβ* expression throughout osteogenic differentiation (0, 1 and 2 wks). (B, left) Enzymatic alkaline phosphatase activity normalized to total protein content after 7d differentiation with or without E2. (B, right) Photometric quantification of Alizarin red staining after 14d differentiation with or without E2. (C) Alkaline phosphatase staining with or without E2 after 7d differentiation. (D) Alizarin red staining with or without E2 after 14d differentiation. (E, left) Expression of early markers of osteogenic differentiation (*Runx2, ALP* and *Col1α*) at 4d differentiation with or without E2. (E, right) *Osteocalcin* expression after 14d differentiation with or without E2. (F) *Bmp2, Bmp4* and *Bmp7* expression at 4d differentiation with or without E2. (G) *Shh, Ihh, Gli1* and *Ptc1* expression at 4d differentiation with or without E2. Values are normalized and significance levels calculated relative to control groups in grey or 0 wk expression levels in blue, photos are representative of random microscopical fields at 20× magnification, N = 3, *P<0.01.

Estrogens have been shown to promote osteogenesis in disparate cell types, both *in vitro* and *in vivo* [33]–[39]. To assess the osteogenic effects of E2 on SMCs, firstly, *ERα* and *ERβ* gene expression were evaluated throughout osteogenic differentiation (0, 1 and 2 wks in osteogenic differentiation medium). As has been reported in other cell types, expression of both *ERα* and *ERβ* were observed to increase overtime in osteogenic medium, (Fig. 4A, N = 3, *P<0.01) [37], [40]. After 7d differentiation, staining and quantification of alkaline phosphatase (ALP) activity was performed as an early marker of osteogenic differentiation (Fig. 4B,C). Results showed that E2 increased both the intensity of ALP staining, and absolute value of enzymatic activity when normalized to total protein content (Fig. 4B,C, N = 3, *P<0.01). Terminal osteogenic differentiation was assessed at 14d differentiation, by Alizarin red staining of bone nodules and colorimetric quantification (Fig. 4B,D, N = 3, *P<0.01). Congruent with ALP activity, Alizarin Red staining demonstrated a dose-dependent increase with E2 addition (Fig. 4B,D). Gene expression of osteogenic specific markers was assessed to verify E2 mediated enhancement of *in vitro* osteogenesis (Fig. 4E). Consistent results were obtained both with early markers *(Runx2, ALP, Col1α1)* and terminal markers of differentiation *(Osteocalcin)*: each showed a significant increase in expression with E2 as compared to osteogenic medium alone (Fig. 4E, N = 3, *P<0.01). Thus, E2 addition to osteogenic medium enhanced SMC osteogenic differentiation.

Bone morphogenetic proteins (BMP)-2, -4 and -7 are members of the BMP family important in osteogenic differentiation [41]. Molecular studies have indicated that E2 may promote osteogenic differentiation in some cell types by promoting transcription of pro-osteogenic bone morphogenetic proteins (BMPs); we sought to investigate whether the same is true in SMCs [38], [42]–[45]. Interestingly, *Bmp2, -4, and -7* expression was observed to increase with E2 addition to medium; a 2–4 fold up-regulation in each cytokine was observed, (Fig. 4F, N = 3, *P<0.01). Hedgehog signaling via either Sonic Hedgehog (Shh) or Indian Hedgehog (Ihh) has been observed to be of importance in both osteogenic and chondrogenic differentiation, *in vitro* and *in vivo* [46]–[50]. We next evaluated gene expression of Hedgehog ligands (*Shh, Ihh*) and downstream signaling elements (*Ptc1, Gli1*) after E2 treatment, by qRT-PCR. Interestingly, and as has been described in other cell types [51], E2 increased transcript abundance for all Hedgehog signaling elements examined, (Fig. 4G, N = 3, *P<0.01). This suggested that the pro-osteogenic effect of E2 in SMCs may be, in part, due to increased transcription of BMP and Hh family members.

### Fulvestrant decreases osteogenic differentiation of PF suture-derived mesenchymal cells

**Figure 5:**
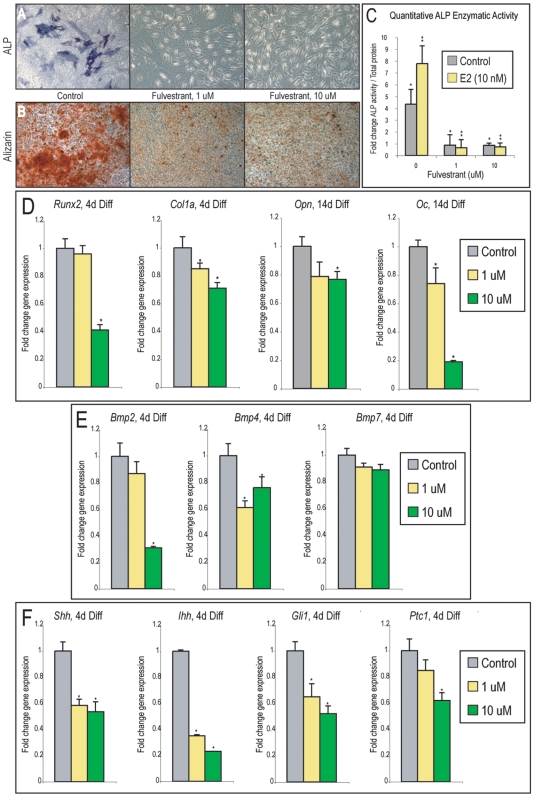
Osteogenic differentiation of PF SMCs with Fulvestrant. (A) Alkaline phosphatase staining with or without Fulvestrant after 7d differentiation. (B) Alizarin red staining with or without Fulvestrant after 14d differentiation. (C) Enzymatic alkaline phosphatase activity normalized to total protein content after 7d differentiation with or without Fulvestrant. (D, left) *Runx2* and *Col1a* expression after 4d differentiation with or without Fulvestrant. (D, right) *Osteopontin* and *Osteocalcin* expression after 14d differentiation with or without Fulvestrant. (E) *Bmp2, Bmp4*, and *Bmp7* expression after 4d with or without Fulvestrant. (F) *Shh, Ihh, Gli1*, and *Ptc1* after 4d differentiation with or without Fulvestrant. Values are normalized and significance levels calculated relative to control groups in grey, photos are representative of random microscopical fields at 20× magnification, N = 3, *P<0.01.

Having shown that E2 positively influences SMC osteogenic differentiation, we next inquired as to whether the converse was true: whether blockade of endogenous estrogen signaling negatively influenced *in vitro* osteogenesis. Fulvestrant, a synthetic pan-ER antagonist, was added to osteogenic culture conditions (Fig. 5). Fulvestrant has been observed to decrease osteogenic differentiation of various bone forming cells *in vitro* [52]–[54]. Staining for ALP and Alizarin red was performed as previously shown (Fig. 5A,B). While SMCs in osteogenic medium alone showed relatively abundant staining, Fulvestrant when added at all concentrations diminished the intensity of staining for both ALP and Alizarin red staining (N = 3, 1–10 uM). Quantification of enzymatic ALP activity was next performed with Fulvestrant, with or without E2 (Fig. 5C, N = 3, * and **P<0.01). Similar to results from biochemical staining, ALP enzymatic activity was significantly reduced with Fulvestrant. Moreover, E2 mediated up-regulation of ALP activity was reversed by addition of Fulvestrant in combination (yellow bars Fig. 5C, N = 3, **P<0.01). Specific gene expression was examined by qRT-PCR, demonstrating a significant decrease in gene markers of early as well as terminal osteogenic differentiation (*Runx2, Col1a, Osteopontin, Osteocalcin*, Fig. 5D, N = 3, *P<0.01). We observed previously that culture with E2 significantly increased a number of pro-osteogenic cytokines, including BMP and Hh ligands (see Fig. 4). Interestingly, we found the converse to be true as well: culture with Fulvestrant resulted in a decrease in transcript abundance for *Bmp2, Bmp4, Shh, Ihh* as well as downstream Hh signaling components (*Gli1, Ptc1*), by qRT-PCR (Fig. 5E,F, N = 3, *P<0.01). Thus, these data suggested that inhibition of endogenous ER signaling impedes *in vitro* SMC osteogenesis, accompanied by a decrease in BMP and Hh signaling.

### 17-β Estradiol increases chondrogenic differentiation of PF suture-derived mesenchymal cells

**Figure 6:**
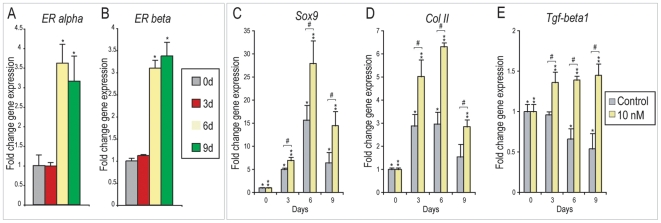
Chondrogenic differentiation of PF SMCs with 17-β Estradiol. (A–B) *ERα* and *ERβ* mRNA expression overtime during *in vitro* chondrogenic differentiation. Both transcripts were observed to increased overtime in culture after 6d differentiation. (C) *Sox9* expression during chondrogenic differentiation with or without E2 (10 nM). (D) *Col II* expression with or without E2 (10 nM). (E) *Tgf-β1* expression with or without E2 (10 nm). Values are normalized and significance levels calculated relative to control groups or 0wk expression in grey or blue, N = 3, *P<0.01.

Estrogen receptors are normally expressed in chondrocyte growth plates and have been shown to positively regulate chondrocyte maturation in various models [55]–[59]. Our previous observations had suggested that in the PF suture as well, estrogen signaling may play a role in chondrogenesis. For example, using immunohistochemistry we previously noted that ER protein expression was strongly present in chondrocytes within the PF suture, (see also Fig. 1E). In addition, the αERKO mouse demonstrated a complete lack of cartilage within the PF suture (see also Fig. 2B,D). Therefore, we next inquired as to the effects of estrogen signaling in the *in vitro* chondrogenic differentiation of SMCs (Fig. 6).

Firstly, *ERα* and *ERβ* expression were evaluated over 9d chondrogenic differentiation by qRT-PCR. Results showed that gene expression of both estrogen receptors increased after 6d in chondrogenic medium, (Fig. 6A,B, N = 3, *P<0.01). We next added E2 to chondrogenic medium and examined gene markers of chondrogenic differentiation (10 nM). Expression of the transcription factor *Sox9*, as well as extracellular matrix component *Col II* were assessed, see (Fig. 6C,D, N = 3, *P<0.01). PF SMCs showed significantly increased expression of both *Sox9* and *Col II* overtime under chondrogenic condition (grey bars Fig. 6C,D, N = 3, *P<0.01). Addition, however, of E2 significantly increased both *Sox9* and *Col II* expression to a greater degree (yellow bars Fig. 6C,D, N = 3, #*P*<0.01). Thus, addition of E2 enhanced markers not only of osteo- but also chondrogenic differentiation of SMCs *in vitro*.

TGF-β1 induces chondrogenic differentiation of multiple cell types *in vitro* and *in vivo* [8], [60], [61]. We examined *Tgf-β1* gene expression upon E2 addition to chondrogenic medium (Fig. 6E). Without E2, *Tgf-β1* gene expression was observed to decreased overtime in chondrogenic culture (Fig. 6E, N = 3, *P<0.01). In contrast, E2 addition led to a significant up-regulation of *Tgf-β1* expression (Fig. 6E, **P<0.01). Thus, the pro-chondrogenic effect of E2 in PF SMCs may in part be due to maintained TGF-β signaling.

### Fulvestrant inhibits calvarial osteogenesis in vivo

**Figure 7:**
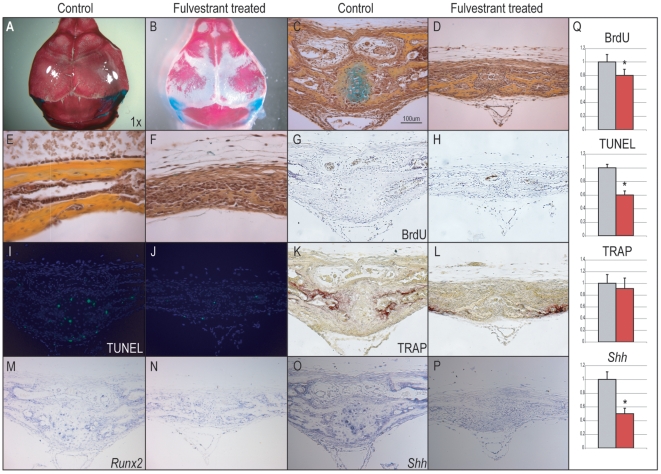
Fulvestrant application to mouse calvaria. (A–B) Control treated and Fulvestrant treated calvariae (N = 6, N = 10 respectively) (A) Dorsal view of p10 bone and cartilage preparation, showing normal calvarial morphogenesis in vehicle treated calvaria. (B) Overhead view of comparable Fulvestrant treated calvaria. A significant delay in mineralization was observed with widened sutures. (C) Pentachrome staining of p10 PF suture sectioned in a coronal plane. Normal developmental timing is observed, including progressive fusion and presence of a cartilage intermediate. (D) Comparable histological section of Fulvestrant treated a p10 PF suture, patency and diminished osteoid is apparent without cartilage. (E–F) Image of mid-frontal bone in control as compared to Fulvestrant treated calvaria, pentachrome stain. Severely diminished osteoid deposition (appearing yellow) is apparent among Fulvestrant treated calvaria. (G–H) BrdU incorporation in control in comparison to treated PF sutures. BrdU positive cells appear brown, while nuclei are counterstained with haemotoxylin. No significant difference was observed between control and Fulvestrant treated sutures (I–J) TUNEL staining in control and Fulvestrant treated PF sutures. Positive cells appear green. (K–L) TRAP staining in control and Fulvestrant treated PF sutures. Positively staining cells appear red. (M–N) *Runx2 in situ* hybridization in control and Fulvestrant treated PF sutures. Positively staining cells appear purple. (O–P) *Shh in situ* hybridization in control and Fulvestrant treated PF sutures. Positively staining cells appear purple. (Q) Quantitative assessments of percentage +BrdU, +TUNEL, or +TRAP stained cells, as calculated using Adobe Photoshop. For quantitative assessments, values are normalized and significance levels calculated relative to control treated groups shown in grey, *P<0.01.

Genetic knockout models suggested that estrogen signaling through ERα was necessary for normal PF suture fusion. We next inquired whether iatrogenic, biochemical perturbation of ER signaling could impede normal suture fusion *in vivo*. The biochemical antagonist Fulvestrant was applied subcutaneously, daily, to the midline of the mouse skull starting at p1 and applied for the following 10 days (10 ul of a 100 uM solution). Various *in vivo* applications of Fulvestrant have resulted in decreased bone volume accompanied by increased bone turnover [62]–[64]. Animals injected with vehicle control appeared grossly and histologically identical to non-manipulated animals and exhibited normal suture morphology (Fig. 7A,C,E). In marked contrast, those animals treated with Fulvestrant showed severely diminished calvarial osteogenesis, accompanied by widely patent sutures (N = 10, Fig. 7B,D,F). Cellular proliferation and cell death were examined in control and Fulvestrant treated calvariae by BrdU and TUNEL staining, respectively (Fig. 7G–J). A slight decrease in cellular proliferation was observed in treated as compared to control specimens. Positive TUNEL staining was sparsely observed in both control and treated specimens; most staining was observed among control specimens in those chondrocytes undergoing apoptosis within the fusing PF suture. Quantification of BrdU and TUNEL staining was performed using Adobe Photoshop, normalized to total cell number, (Fig. 7Q). This suggested that the phenotype observed with Fulvestrant treatment was not likely due to a direct change in either cellular proliferation or cell viability. We inquired as to whether Fulvestrant injection resulted in widespread osteoclastogenesis and concomitant bone turnover; this was assessed in treated and control calvariae by TRAP staining. Again, no differences were observed, suggesting that increased bone turnover could not explain the phenotype of Fulvestrant treated calvaria (Fig. 7K,L,Q). Finally, gene expression levels of *Runx2* and *Sonic hedgehog (Shh)* were evaluated by *in situ* hybridization (Fig. 7M–P). As previously observed in the αERKO skull, Fulvestrant treatment resulted in diminished expression for *Runx2* and *Shh*. Although *Runx2* expression was not appreciably diminished, a slight difference in expression pattern was observed.

In summation, in this study we sought to correlate estrogen signaling with mouse cranial suture fusion, a process of endochondral ossification [4]. We found that *ERα* gene transcript abundance temporally coincides with PF suture fusion. Moreover, immunohistochemistry detected ER protein primarily within osteocytes and chondrocytes in cranial suture mesenchyme. Via analysis of ER knockout mice, functional ERα but not ERβ was found to be necessary for normal suture fusion. *In vitro* cell culture of suture-derived mesenchymal cell (SMCs) suggested that 17-β estradiol (E2) enhanced both osteogenic and chondrogenic differentiation within the PF suture. Finally, *in vivo* blockade of ER signaling in the developing calvaria via Fulvestrant inhibited suture fusion and led to severely diminished calvarial osteogenesis.

## Discussion

Estrogens have been long considered of importance in bone and cartilage biology, as well as skeletal development, maturation and healing [22]. Therefore, natural and synthetic estrogens have been studied in basic osteochondroblast biology, as well as in clinical problems as bony non-union, osteoporosis, and osteoarthritis [33], [57], [65], [66]. In addition, functional estrogen signaling has been found to be necessary for developmental growth plate fusion [29]–[31]. This study addressed a related and straight-forward question: to what extent does estrogen signaling play a role in the development and fusion of cranial sutures? To study this, we chose an animal model of physiologic suture fusion: the mouse posterofrontal suture.

To date, we are only aware of one other group linking cranial suture biology with sex hormone signaling [12], [13]. Lin *et al.* in two publications examined androgen signaling in cranial sutures. They showed that androgen receptors are strongly expressed in fetal calvaria and dura mater, that cell culture with the male sex hormone dihydrotesterone (DHT) increased osteoblastic gene markers, and sagittal suture organ cultures aberrantly fused with DHT [12], [13]. As in our publication, they found similar patterns of receptor expression between male and female mice. Other groups have similarly noted comparable ER expression levels in bony tissue between the sexes [15], [16]. Our findings and others bring to the fore puzzling questions regarding the specificity of sex hormones in cranial suture biology. Firstly, despite the clear effects of hormone signaling on cranial suture fusion, how are sutures derived from male and female mice morphologically identical? Next, does genetic deficiency in sex hormone receptors affect both male and female derived sutures equivalently, or disparately? Interestingly, the severity of the appendicular skeletal phenotype of ERKO mice differs by sex [26], [27]; it remains unclear if these differences extend to the craniofacial skeleton. Our findings by quantitative RT-PCR, immunohistochemistry and cell culture, however, suggest that estrogens play a relevant role in suture developmental biology regardless of sex. Analogous findings are seen clinically as patients with either aromatase deficiency, ERα deficiency, or androgen insensitivity syndrome, have osteopenic skeletons, suggesting a need for both androgen and estrogen receptor signaling for maintenance of bone density. More detailed study however, particularly *in vitro* cell culture of male or female derived cells, is necessary to pursue these conjectures.

Our analysis of ERα and ERβ gene expression and ERKO mice suggest that ER receptors are of unequal importance in cranial suture biology. Firstly, *ERα* but not *ERβ* expression peaked during suture fusion. In fact, *ERβ* expression demonstrated little change in either fusing (PF) or patent (SAG) sutures throughout early postnatal development. In addition, the PF suture of the αERKO mouse exhibited a delayed suture phenotype. Moreover, thorough analysis of the βERKO mouse found normal suture morphogenesis, including normal developmental timing of PF suture fusion. Thus, these data suggest that ERα is of primary, and ERβ secondary, importance in cranial suture biology. Other data support this hypothesis: we noted that ERα is expressed to a greater degree in the calvaria than ERβ. In our hands, semi-quantitative PCR and Western blot both showed this to be true: robust bands for *ERα* expression were seen, while less intense bands were discernable for *ERβ* expression (Fig. S3A,B).

We observed that application of the pan-ER antagonist Fulvestrant to the developing mouse calvaria resulted in a substantial decrease in cranial osteogenesis. While this iatrogenic defect mimicked the αERKO mouse suture phenotype, it was relatively more severe. How does one reconcile this difference in severity of phenotype produced by biochemical blockade versus genetic deficiency? TUNEL staining excluded cell death among Fulvestrant treated calvaria. One possibility is that ER genetic deficiency allows time for compensatory mechanisms to develop, allowing osteoblastic differentiation and mitigating the αERKO phenotype. In contrast, biochemical ER blockade via Fulvestrant injection represents a sub-acute insult to the developing skull, whereby no compensation can ensue. Another possibility is that intact signaling via ERβ may partially mitigate the αERKO phenotype. Nevertheless, perturbation of ER signaling via both methods resulted in persistent PF suture patency, and indicated estrogen/ERα signaling as necessary for normal mouse suture fusion.

In an effort to more fully delineate the importance of ERα vs ERβ signaling, our laboratory has utilized synthetic estrogen agonists specific for either ERα or ERβ in cell culture (propyl pyrazole triol, PPT, and diarylpropionitrile, DPN, respectively). Interestingly, our *in vitro* analysis suggests that both specific agonists up-regulate osteochondrogenic gene expression in SMCs (including *Runx2* and *Sox9*, see Fig. S3C,D). How again does this reconcile with the apparent suture phenotype in the αERKO but not βERKO mouse? From these data we speculate that estrogen/ERα signaling may be of principal importance in cranial suture biology owing to the comparative abundance of receptor expression in the developing cranial suture complex (Fig. S3A,B). Conversely, the relatively small expression level of ERβ, as demonstrated on both gene and protein levels, may belie its importance. Moreover, ERβ expression levels were observed to remain unchanged in the αERKO mouse, which argues against a functional compensation. More study utilizing specific ER agonists *in vivo* may further bring to light the relative importance of ERα and -β.

Most studies in the field of cranial suture biology have stressed the relative importance of dura mater in determining fate and timing of suture development. This includes locoregional production of FGF-2, TGF-β1, the BMP antagonist Noggin, among others [8], [10], [67], [68]. These paracrine factors are hypothesized to work locally on the overlying suture mesenchyme, promoting differentiation and ultimately suture fusion [3]. In this conceptual, regulatory framework, how might an endocrine hormone influence suture fusion? Our study has identified estrogen responsive cells by localizing ER expression patterns, but does not indicate a source for estrogen ligands, whether it be locoregional or distant. Studies in long bones, however, suggest that production of estrogens by adjacent periosteum affects growth plate maturation [69], [70]. We speculate that dura mater, a known source of paracrine factors integral to suture fusion, may analogously be a source of regional estrogen production. More study is needed to probe this conjecture. Alternatively, those peptide growth factors known to be localized to PF-associated dura mater (including TGF-β and IGF-1) have been shown to exert transcriptional effects in an estrogen receptor-dependent manner [71]–[73]. Thus, known local paracrine factors may exert cellular and morphologic effects on the overlying suture in an ER-dependent manner, either in the absence or in synergism with E2.

Despite the as yet unclear mechanism whereby E2 may selectively be involved in PF suture fusion, our *in vitro* studies in suture-derived mesenchymal cells (SMCs) suggest a transcriptional relationship between E2 and other cytokines known to play a role in suture biology. For example, transcription of a number of pro-osteogenic BMPs was positively regulated by E2 in PF SMCs *in vitro* during osteogenic differentiation (Fig. 4F). Moreover, both Sonic and Indian Hedgehog, known important players in osteoblast and chondrocyte differentiation, were up-regulated by E2 as well (Fig. 4G). In addition, transcription of *Tgfβ1* was increased with E2 in PF SMCs (Fig. 6E). Both BMP and TGF-β signaling are associated with and necessary for physiologic cranial suture fusion [8], [10], [11], [74], and in our *in vitro* model system were positively regulated by E2. Moreover, abundant data in the literature suggest a positive intersection between estrogen and IGF signaling [72], [73], [75], [76], another peptide molecule of known importance in cranial suture fusion [77]–[79]. We explored this possibility, however E2 was not observed to significantly effect IGF gene expression in SMCs *in vitro* (data not shown). More *in vivo* correlates must be sought, but these data potentially implicate estrogen signaling in a cascade of known molecular mediators in cranial suture fusion.

Especially pertinent to our discussion is the possible relationship between E2/ERα signaling and Hedgehog (Hh) signaling in cranial sutures. Interestingly, while the WT posterofrontal cranial suture shows abundant staining for hedgehog ligands, the αERKO PF suture did not (Fig. 2G,H). Moreover, Hedgehog signaling was dynamically regulated by E2 or Fulvestrant in SMCs *in vitro* (Fig. 4,5). It is known that mice with genetic deficiency in Ihh have severe skeletal defects, including severely shortened limbs due to a defect in endochondral ossification [50]. Moreover, the Ihh null mouse has a cranial suture phenotype with reduced calvarial bone size, a phenotype which may have similarities to the αERKO calvaria, although this has not been well studied [50]. Hedgehog signaling has been shown to be up-regulated by estrogen signaling [51], [80]. To our knowledge, this is the first publication in which estrogen signaling has been shown to positively regulate Hedgehog signaling in bone-forming cells of any type. Moreover, preliminary data from our laboratory suggests that increased Hedgehog signaling (via the addition of N-terminal Sonic Hedgehog to osteogenic medium) significantly enhances the osteogenic differentiation of SMCs in a similar fashion to E2 (data not shown). The potential cooperative relationship between E2 and Hh signaling will be the topic of future research.

Important limitations to this study exist toward the extrapolation to the clinical entity of craniosynotosis. The posterofrontal suture is an example of physiologic suture fusion, and thus has parallels to the human metopic suture, in both, there exists a cartilage intermediate preceding closure [4], [5]. However, chondrogenesis has not been shown to precede clinical craniosynostosis, whether syndromic or sporadic. Moreover, a cartilaginous intermediate does not develop in any other sutures of the mouse skull. Therefore, while this study has immediate applicability to normal calvarial skeletogenesis and programmed suture fusion, the relevance of these data to clinical, pathologic suture fusion remains as yet unclear. ER expression patterns in human synostotic sutures may yield fruitful insight into the possible connection between pathologic suture fusion and estrogen signaling.

In conclusion, the role of estrogen signaling is of newly recognized importance in suture biology. ER gene expression is associated with physiologic cranial suture fusion. Intact ERα signaling is necessary for normal mouse cranial suture fusion. Finally, culture of PF suture-derived cells suggests that estrogen signaling may positively regulate both osteoblast and chondrocyte differentiation within the suture complex.

## Materials and Methods

### Chemicals, supplies and animals

Phenol red-free Dulbecco's Modified Eagles Medium (DMEM) and phenol red-free penicillin/streptomycin were purchased from GIBCO Life Technologies (Carlsbad, CA). Fetal bovine serum (FBS) was purchased from Omega Scientific (Tarzana, CA). All cell culture supplies were purchased from Corning Inc. (San Mateo, CA). RNAeasy kits were purchased from Qiagen Sciences (Valenica, CA), while other RNA reagents were purchased from Applied Biosystems, (Foster City, CA). Unless otherwise specified, all other chemicals were purchased from Sigma-Aldrich (St. Louis, MO).

All experiments followed protocols approved by the Animal Facilities of Stanford University and the NIEHS/NIH. CD-1 mice were purchased from Charles Rivers Laboratories (Wilmington, MA). Male αERKO and βERKO mice on a C57BL/6J background and wild-type littermates were provided from the NIEHS, and were genotyped as previously described [81]. Animals were housed in a light and temperature controlled facility and given food *ad libitum*. For all experiments, the first day of life was considered the first day after birth.

### Whole mount bone and cartilage preparations

Whole calvariae denuded of skin were fixed overnight in 100% ethanol (postnatal days 4–19, N = 3–5 per time point from both male and female mice). Calvariae were then stained with an Alcian blue solution in 30% acetic acid/70% ethanol to visualize cartilage. Samples were then washed in 0.5% potassium hydroxide, stained with an 0.01% Alizarin Red S solution in 0.5% potassium hydroxide to visualize mineralized bone, washed in PBS, and cleared in a series of graded glycerol. Pictures were taken at 1×–1.6× magnification utilizing the Leica digital imaging system; specimens were stored at room temperature.

### Preparation of tissues for histology

Whole calvariae denuded of skin were fixed overnight in 0.4% paraformaldehyde in PBS at 4°C (postnatal days 4–30, N = 2–3 per time point). Tissue was taken from equal numbers of male and female mice. PF and SAG sutures with flanking calvarial bones were dissected, tissue was decalcified in 19% ethylenediaminetetracetic acid for 2–14 days at 4°C, dehydrated through graded ethanol, and paraffin embedded. Coronal sections through PF and SAG sutures of 5 micron width were mounted on Superfrost plus slides (Fisher Scientific, Pittsburg, PA), and dried overnight at 37°C. Approximately 200 sections were obtained from each skull; slides were stored at room temperature.

### Histological staining and immunohistochemistry

Every 5^th^ slide of PF and SAG sutures were stained with routine haematoxylin and eosin. Select slides were stained with pentachrome stain to visualize both bone and cartilage, which appear yellow and blue/green, respectively. Cellular proliferation was assessed by bromodeoxyuridine (BrdU) and PCNA immunohistochemical staining according to the manufacturer's instructions (Zymed, South San Francisco, CA). A haematoxylin counterstain was employed. BrdU labeling reagent was administered 2 hrs prior to mouse sacrifice. Cell death was examined by an in situ cell death detection kit per the manufacturer's instructions (Roche Applied Science, Indianapolis, IN). A DAPI counterstain was employed. Bone turnover was assayed by tartrate resistant acid phosphatase staining (TRAP staining) according to the manufacturer's recommendations.

Immunohistochemistry was performed on select slides for ERα and ERβ. Slides were deparaffinized and rehydrated. Endogenous peroxidase activity was quenched with 3% hydrogen peroxide in methanol; slides were blocked with 10% goat serum in PBS. Antibodies used included rabbit polyclonal anti-ERα, (1∶80 in dilution, Santa Cruz Laboratories, Santa Cruz, CA), or rabbit polyclonal anti-ERβ (1∶2 dilution, BioGenex, San Ramon, CA), and were suspended in 1% rabbit serum. Appropriate biotinylated secondary antibodies were used in 1∶1000 dilution (Vector Laboratories, Burlingame, CA). The Vectastain ABC system (Vector Laboratories, Burlingame, CA) was used according to the manufacturer's instructions. Visualization was with diaminobenzidine solution (Zymed Laboratories, South San Francisco, CA). Slides without primary antibody were used as a negative control. No less than 5 slides were stained for each antibody, per time point. To account for possible gender variability, for each time point immunohistochemistry was performed on equal numbers of slides taken from both male and female mice. Representative photographs were taken at 20×–40× magnification (Zeiss Axioplan, Thornwood, NY).

*In situ* hybridization for *Runx2, Ihh* and *Shh* was performed as previously described [82]. Antisense riboprobe was transcribed in the presence of Dig-11-UTP (Roche). Sections were incubated for 65°C for 12 hrs in hybridization buffer (Ambion, Austin TX) containing riboprobe at ∼1 ug/mL. Slides were blocked with 10% sheep serum, 2% Boehringer-Mannheim Blocking Reagent (Roche) and levamisole, and developed using NBT and BCIP for color.

To assess quantitative differences in staining, Adobe photoshop was utilized. Pixel number of positively stained cells for BrdU, TUNEL, TRAP or Shh were quantified from 5 random high-powered fields using the magic want tool. Values were divided by the pixel number of DAPI staining in the same images. Means and standard deviations were calculated.

### Quantitative Assesment of Suture Fusion

To assess degree of suture fusion, the entirety of each suture was sectioned. Every 5^th^ section was stained with H&E (equivalent to one section per 25 microns), and was evaluated by two independent observers. Fusion was defined as bony bridging between osteogenic fronts of the suture, and was considered a binary variable (1 = fusion, 0 = patent). In the case of interobserver disagreement, a third party made the determination. To calculate percentage fusion, the number of individual slides showing a fused suture was divided by the total number of stained slides. This method was taken with adaptation from our previous publication [74].

### Quantification of gene expression in cranial sutures in vivo

Total ribonucleic acid (RNA) was isolated from PF and SAG suture complexes at stratified postnatal time points (postnatal days 4, 7, 10, 13, 16, 19 - points preceding, during, and after fusion of the PF suture; N = 10 per time point of mixed gender). Calvariae were dissected in cold, sterile phosphate-buffered saline (PBS). Pericranium was meticulously removed with fine-tipped forceps under a dissecting microscope (Dumont #55 Forceps, Fine Science Tools, Foster City, CA). PF and SAG sutures were then isolated with 500 micron bony margins on either side as well as underlying dura mater; dissections were based on previously established anatomic landmarks [4]. Sutures from each time point were pooled, snap-frozen in liquid nitrogen, homogenized with a pestle, and purified using the RNeasy Mini Kit. DNase treatment was performed with the DNA-*free* kit according to the manufacturer's instructions (Ambion, Austin, TX). One ug RNA was reverse transcribed using TaqMAN® Reverse Transcription Reagents. Quantitative real-time polymerase chain reaction (qRT-PCR) was performed with *Power* Sybr green detection on a 7900HT Sequence Detection System according to the manufacturer's instructions. Specific primers for the genes examined were designed based on their PrimerBank sequence (http://pga.mgh.harvard.edu/primerbank). Primer sequences are shown in **Table 1**. PCR products were first run on a 2% agarose gel to confirm the appropriate size and specificity. Levels of gene expression were determined by normalizing to their *GAPDH* values. All reactions were performed in triplicate.

**Table 1:** Quantitative PCR Genes and Primer Sequences.

Gene Name	NCBI GeneID	Forward primer sequence (5′ to 3′)	Reverse primer sequence (5′ to 3′)
Alkaline phosphatase	11647	GTTGCCAAGCTGGGAAGAACAC	CCCACCCCGCTATTCCAAAC
Bmp2	12156	GGGACCCGCTGTCTTCTAGT	TCAACTCAAATTCGCTGAGGAC
Bmp4	12159	TTCCTGGTAACCGAATGCTGA	CCTGAATCTCGGCGACTTTTT
Bmp7	12162	CGATACCACCATCGGGAGTTC	AAGGTCTCGTTGTCAAATCGC
Collagen Type IαI (ColIαI)	12814	AACCCGAGGTATGCTTGATCT	CCAGTTCTTCATTGCATTGC
Collagen Type II (Col II)	12824	TCCAGATGACTTTCCTCCGTCTA	CAGGTAGGCGATGCTGTTCTTAC
Estrogen Receptor α (ERα)	13982	CCTCCCGCCTTCTACAGGT	CACACGGCACAGTAGCGAG
Estrogen Receptor β (ERβ)	13983	CTGTGATGAACTACAGTGTTCCC	CACATTTGGGCTTGCAGTCTG
GAPDH	14433	AGGTCGGTGTGAACGGATTTG	TGTAGACCATGTAGTTGAGGTCA
Gli1	14632	TCGACCTGCAAACCGTAATCC	TCCTAAAGAAGGGCTCATGGTA
Indian hedgehog (Ihh)	16147	GCTTCGACTGGGTGTATTACG	GCTCGCGGTCCAGGAAAAT
Osteocalcin (Oc)	12096	GGGAGACAACAGGGAGGAAAC	CAGGCTTCCTGCCAGTACCT
Osteopontin (Opn)	20750	TAGCTTGGCTTATGGACTGAGG	AGACTCACCGCTCTTCATGTG
Patched (Ptc1)	19206	GCCAAGCCCTAAAAAAAT	ACCACAATCAATCTCCTG
Runx2	12393	CGGTCTCCTTCCAGGATGGT	GCTTCCGTCAGCGTCAACA
Sonic hedgehog (Shh)	20423	AAAGCTGACCCTTTAGCCTA	TTCGGAGTTTCTTGTGATCTTCC
Sox9	20682	TACGACTGGACGCTGGTGC	TTCATGGGTCGCTTGACGT
Tgfβ1	21803	AACAATTCCTGGCGTTACCTT	TCCTTCCACAGTATGCTCGTA

### Protein quantification in whole calvariae in vivo

Protein was isolated from the whole calvaria of p10 mouse skulls after meticulous dissection of pericranium, dura mater and ligamentous attachments. 40 ug of total protein extracted with radioimmunoprecipitation assay (RIPA) buffer (containing 50 mM Tris-HCl pH 7.5, 150 mM NaCl, 5% Glycerol, 1 mM EDTA, 1% NP-40, 0.1% SDS and 0.25% Na-deoxycholate) was separated on 7.5% sodium dodecyl sulfate-polyacrylamide gel electrophoresis, transferred to polyvinylidene fluoride membranes, and blocked with 5% milk/Tris-buffered saline-T for 2 hours. Protein detection was performed with primary antibodies against ERα (1∶1000 dilution), ERβ, and β-actin (1∶5000 dilution; Lab Vision, Fremont, CA) in 5% milk/Tris-buffered saline-T overnight at 4°C. Blots were then incubated with the corresponding horseradish peroxidase-linked secondary antibodies (1∶10,000 dilution; BD Pharmingen, San Jose, CA) for 1 hr at room temperature. Blots were developed with ECL detection reagent (Amersham, United Kingdom) and exposed for 1 to 10 minutes using Biomax-MS film (Eastman Kodak, Rochester, NY).

### Tissue harvest and primary cell culture

For all *in vitro* experiments, PF suture-derived mesenchymal cells (SMCs) were harvested from 200 four-day-old CD1 mice via non-enzymatic primary cell harvest as previously described [7], [8], [32], [83]. PF sutures were dissected with 500 um bony margins, meticulously stripped of all pericranial and dural tissues, and plated in 100-mm tissue culture dishes (endocranial surface was placed flush to the culture dish, approximately 10 sutures per plate). Explants were cultured in growth medium containing DMEM, 10% FBS, 100 IU/ml of penicillin/streptomycin, and maintained at 37°C in an atmosphere of 5% CO_2_. Phenol red-free culture medium was used in all assays to avoid its estrogenic effects. Over seven days in culture, SMCs were allowed to migrate from suture explants; cellular yield was approximately 100,000 cells per suture explant. SMCs were passaged by trypsinization; passage one SMCs only were used for all experiments.

### Cellular proliferation assays

The growth of SMCs in response to 17-β estradiol (E2) or Fulvestrant was compared by BrdU incorporation assays as previously described (N = 6) [32]. Cells were seeded in 96-well plates at a density of 1,000 cells/well and were treated with growth medium supplemented with E2 (0.1–100 nM), Fulvestrant (0.1–10 uM), or vehicle as a control (10 ug/ml bovine serum albumin or 0.1% dimethyl sulfoxide, respectively). At 3d and 6d growth, BrdU incorporation assays were performed according to the manufacturer's instruction (Roche Applied Science, Indianapolis, IN). Labeling with BrdU was performed for a period of 8 hrs. Means and standard deviations were calculated.

### Osteogenic differentiation and assessments

Cells were plated in 24-well plates at a density of 10,000 cells/well, and were treated with osteogenic differentiation medium (ODM) containing DMEM, 10% FBS, 100 µg/ml ascorbic acid, 10 mM β-glycerophosphate, 100 IU/ml penicillin/streptomycin as previously described [32]. ODM was supplemented with E2 (1–100 nM), Fulvestrant (0.1–10 uM), the ERα specific agonist propyl pyrazole triol (PPT, 1–100 nM), ERβ specific agonist diarylpropionitrile (DPN, 1–100 nM), or with vehicle as a control (Sigma-Aldrich, St. Louis, MO).

To assess early osteogenic differentiation, alkaline phosphatase (ALP) staining and quantification was performed after 7d differentiation as previously described [32]. For staining, cells were fixed with a 60% acetone, 40% citrate solution, and stained with a diazonium salt with 4% napthol AS-MX phosphate alkaline solution. Alkaline phosphatase positive cells were stained purple. For ALP quantification, protein was isolated in RIPA buffer. The alkaline phosphate activity was assayed by measuring the *p*-nitrophenol formed from the enzymatic hydrolysis of *p*-nitrophenylphosphate. Experiments were performed in triplicate wells; means and standard deviations were calculated.

After 14d osteogenic differentiation, Alizarin red S staining was performed to detect extracellular mineralization as previously described [32]. Briefly, cells were fixed in 100% ethanol and stained with a 0.2% Alizarin red S solution. The red staining represents calcium deposits on differentiated cells.

Finally, total RNA was isolated from SMCs at 2, 4, 7, and 14d of osteogenic differentiation. Gene expression of the transcription factor *Runx2*, as well as other markers of osteogenesis *(Alkaline phosphatase*, *Collagen Type 1α, Osteopontin*, *Osteocalcin, Bone morphogenetic protein-2, -4, -7, Sonic Hedgehog, Indian Hedgehog, Gli1, Ptc1, Sex determining region Y-box 9 (Sox9), ERα, ERβ)* was evaluated by quantitative RT-PCR.

### Chondrogenic differentiation and assessments

Cells were plated in 12-well plates at a high density of 70,000 cells/well; SMCs were treated with chondrogenic differentiation medium containing DMEM, 1% FBS, 37.5 ug/ml ascorbate-2-phophate, ITS premix (BD Biosciences, Franklin Lakes, NJ), and 100 IU/ml penicillin and streptomycin as previously described [7], [8]. E2 (10 nM) or vehicle as a control was supplemented to chondrogenic medium. Medium was supplemented every three days. At 3, 6, and 9d of differentiation, RNA was isolated and gene markers of chondrogenesis were examined by real-time PCR *(Sox9, Collagen Type II, TGF-β1, ERα, ERβ)*. Primer sequences are listed in **Table 1**.

### In vivo Fulvestrant application and assessments

To test the necessity of estrogen signaling in the suture fusion, the synthetic pan-ER antagonist Fulvestrant was applied *in vivo*. Starting at birth, 10 ul of a 100 uM solution Fulvestrant (in a suspension containing 0.9% normal saline, 0.01% DMSO) was injected subcutaneously, daily, directly overlying the midline sutures. Vehicle (0.9% normal saline, 0.01% DMSO) was injected in littermates as a control. Skulls were harvested at p10 for analysis by whole mount bone and cartilage preparations, as well as serial coronal sections for histology.

### Statistical analysis

Means and standard deviations were calculated from numerical data, as presented in the text, figures, and figure legends. In graphs, columns represent means whereas error bars represent one standard deviation. Statistical analysis was performed using the ANOVA two-factor with replication when more than two factors were compared. In supplement, the Welch's two-tailed *t*-test was used when standard deviations between groups were unequal. *P≤0.01 was considered to be significant.

## Supporting Information

Figure S1 Gross morphology of PF and SAG sutures. Whole mount bone (red) and cartilage (blue) preparations of mouse calvaraie, ages postnatal day (p)4 through 19. The posterofrontal (PF) suture lies anterior (above in this orientation), while the sagittal (SAG) suture lies posterior (below). (A) At p4, both PF and SAG sutures are widely separated. (B–E) Islands of bony bridging are observed within the PF suture (p7–16). (F) By p19 (bottom right), the PF suture is largely fused. Note that tongues of cartilage (stained blue) are observed in the early postnatal skull base (p4, p7). Degree of red hue generally represents thickness of mineralized bone, with the exception of the area of the coronal (COR) suture, where this represents overlap of one calvarial bone on another; photographs are taken at 1.6× magnification.
(3.44 MB TIF)

Figure S2 Histological morphology of PF and SAG sutures. Pentachrome staining of coronal sections through PF and SAG sutures, p4–p19. Osteoid appears yellow, while glycosaminoglycan in cartilage appear blue/green. In the PF suture (first and third columns), a cartilaginous intermediate is apparent from p7 to p13 located on the endocranial aspect of the suture (C–F,M,N). From p16 onwards, osseous fusion of the PF suture can be observed (O–R). In contrast, an undifferentiated cellular mesenchyme is observed at all time points in the SAG suture with maintenance of suture patency, (second and fourth columns). Photographs are taken from the anterior aspect of PF and SAG sutures, and are at 20× and 40× magnification.
(7.50 MB TIF)

Figure S3 Comparison of Estrogen Receptor Expression and Stimulation (A) Expression of ERα and ERβ within the p10 mouse skull by semi-quantitative PCR at 30 cycles. ERα is expressed to a greater degree than is ERβ. (B) Expression of ERα and ERβ within the p10 mouse skull by western blot. Experiments were performed in triplicate; mouse uterine tissue was used as a positive control. (B–C) PF SMCs were cultured with or without the ER specific agonists PPT or DPN (ERα and ERβ specific agonists, respectively). Runx2 and Sox9 expression was evaluated after 48 hrs. Results showed that both PPT and DPN significantly and dose-dependently increased Runx2 and Sox9 expression in PF SMCs. No difference was observed between ERα and ERβ specific agonists. Values are normalized and significance levels calculated relative to control groups, N = 3, *P<0.01.
(5.17 MB TIF)
