# Supplementary figures and images for: Estrogen/Estrogen Receptor Alpha Signaling in Mouse Posterofrontal Cranial Suture Fusion
Source: PLoS One. 2009 Sep 22;4(9):e7120. doi: 10.1371/journal.pone.0007120 (PMC2743190; doi:10.1371/journal.pone.0007120)

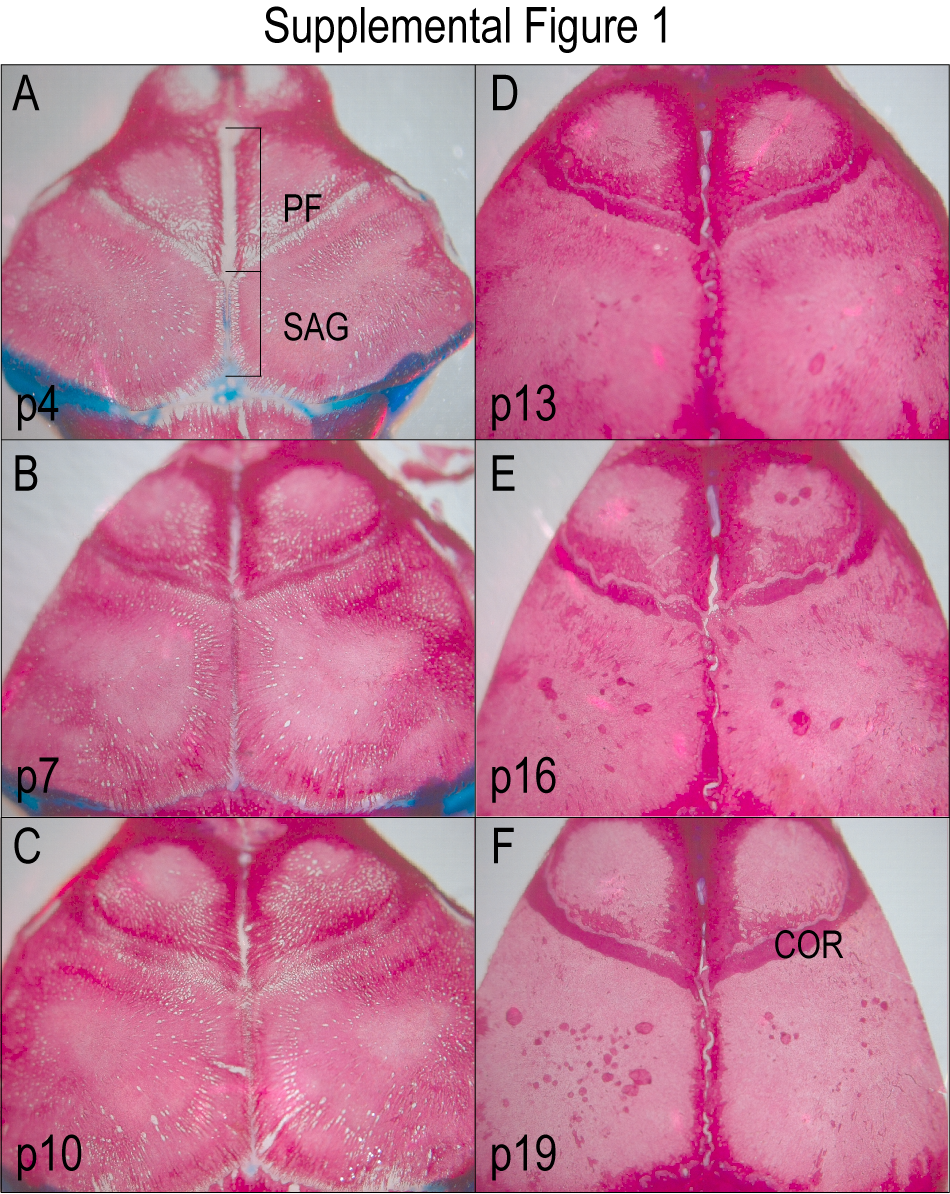

Supplement: Figure S1 — Gross morphology of PF and SAG sutures. Whole mount bone (red) and cartilage (blue) preparations of mouse calvaraie, ages postnatal day (p)4 through 19. The posterofrontal (PF) suture lies anterior (above in this orientation), while the sagittal (SAG) suture lies posterior (below). (A) At p4, both PF and SAG sutures are widely separated. (B–E) Islands of bony bridging are observed within the PF suture (p7–16). (F) By p19 (bottom right), the PF suture is largely fused. Note that tongues of cartilage (stained blue) are observed in the early postnatal skull base (p4, p7). Degree of red hue generally represents thickness of mineralized bone, with the exception of the area of the coronal (COR) suture, where this represents overlap of one calvarial bone on another; photographs are taken at 1.6× magnification. (3.44 MB TIF) [file pone.0007120.s001.tif]

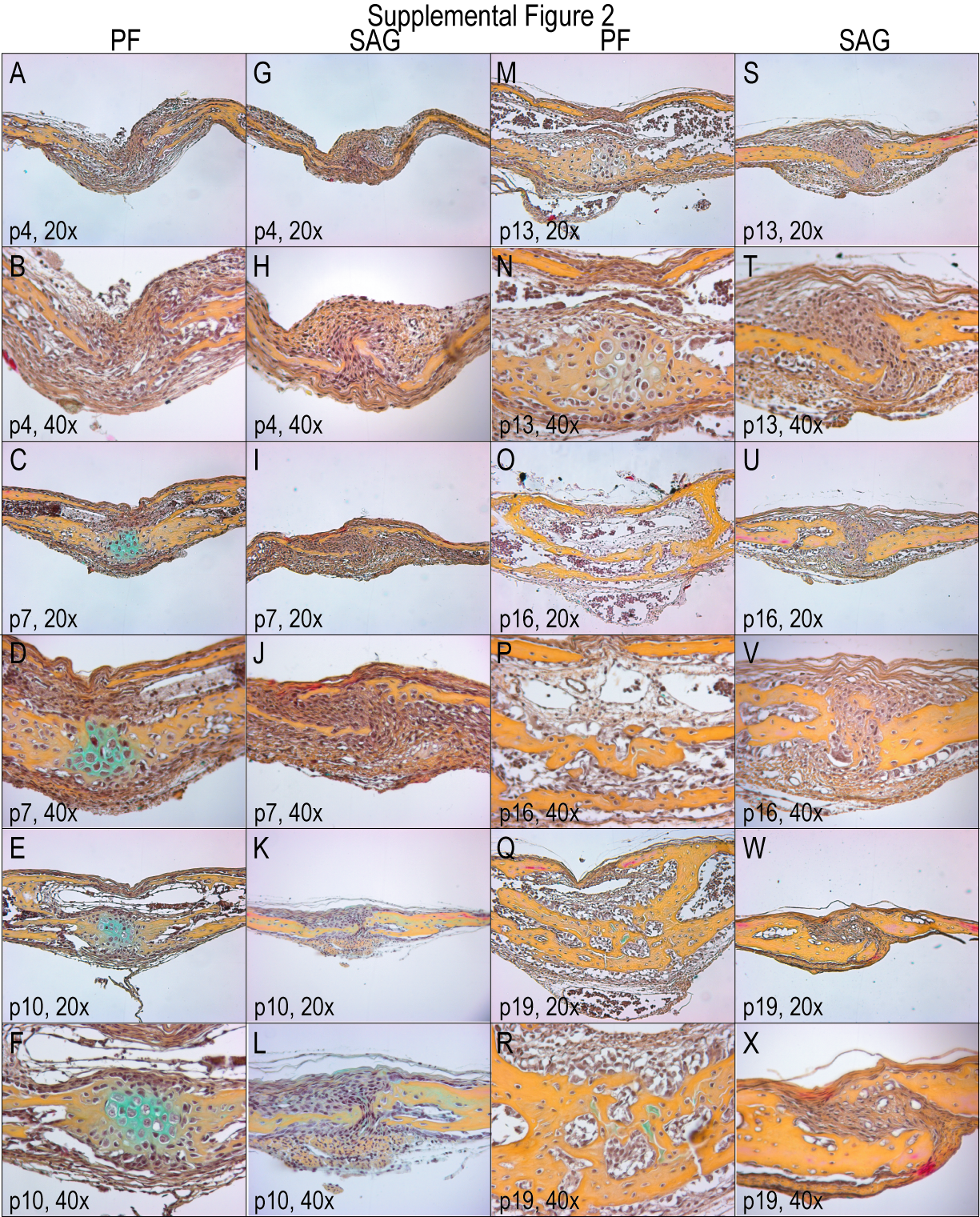

Supplement: Figure S2 — Histological morphology of PF and SAG sutures. Pentachrome staining of coronal sections through PF and SAG sutures, p4–p19. Osteoid appears yellow, while glycosaminoglycan in cartilage appear blue/green. In the PF suture (first and third columns), a cartilaginous intermediate is apparent from p7 to p13 located on the endocranial aspect of the suture (C–F,M,N). From p16 onwards, osseous fusion of the PF suture can be observed (O–R). In contrast, an undifferentiated cellular mesenchyme is observed at all time points in the SAG suture with maintenance of suture patency, (second and fourth columns). Photographs are taken from the anterior aspect of PF and SAG sutures, and are at 20× and 40× magnification. (7.50 MB TIF) [file pone.0007120.s002.tif]

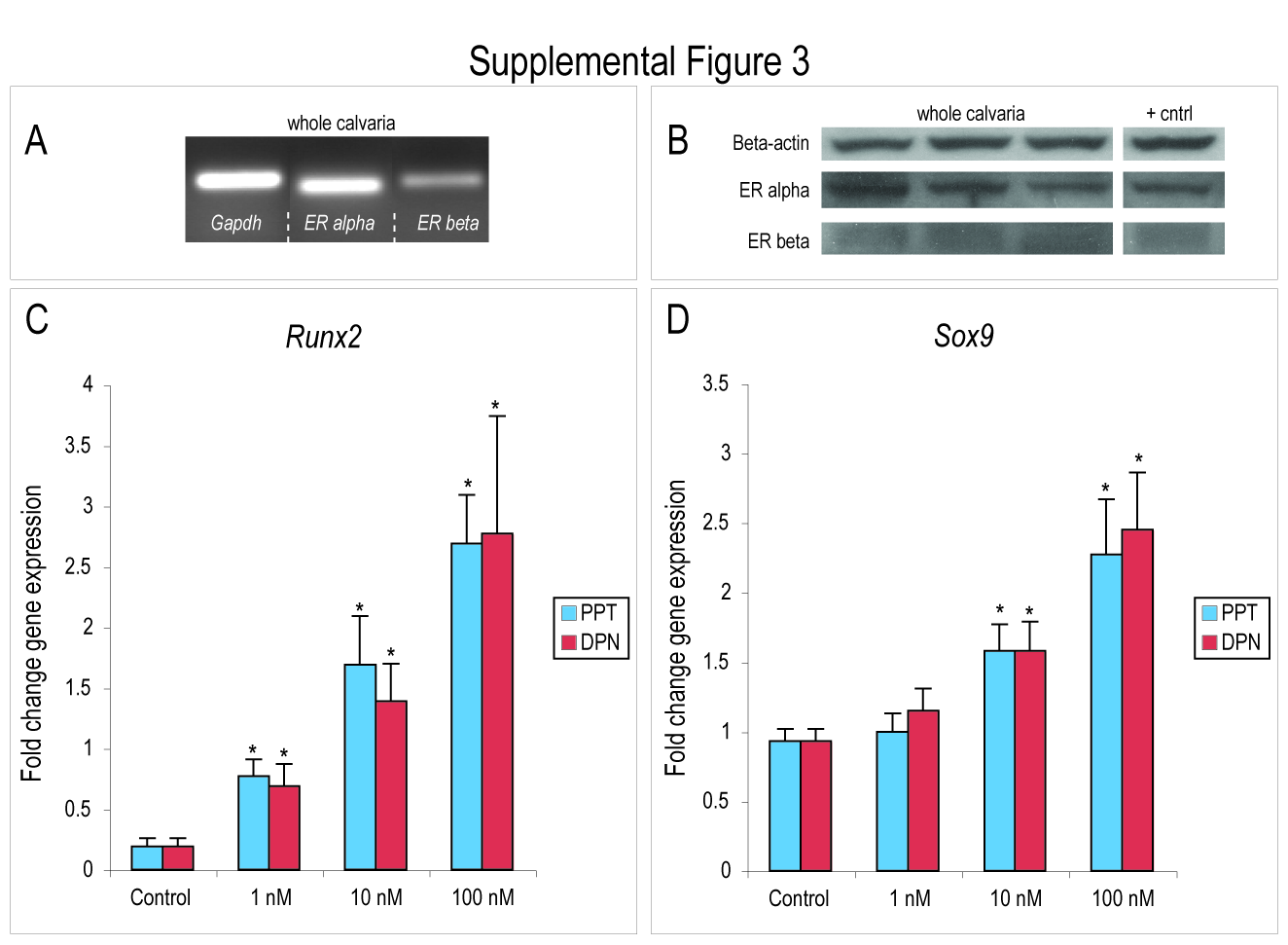

Supplement: Figure S3 — Comparison of Estrogen Receptor Expression and Stimulation (A) Expression of ERα and ERβ within the p10 mouse skull by semi-quantitative PCR at 30 cycles. ERα is expressed to a greater degree than is ERβ. (B) Expression of ERα and ERβ within the p10 mouse skull by western blot. Experiments were performed in triplicate; mouse uterine tissue was used as a positive control. (B–C) PF SMCs were cultured with or without the ER specific agonists PPT or DPN (ERα and ERβ specific agonists, respectively). Runx2 and Sox9 expression was evaluated after 48 hrs. Results showed that both PPT and DPN significantly and dose-dependently increased Runx2 and Sox9 expression in PF SMCs. No difference was observed between ERα and ERβ specific agonists. Values are normalized and significance levels calculated relative to control groups, N = 3, *P<0.01. (5.17 MB TIF) [file pone.0007120.s003.tif]
